# Evaluation of immediate and delayed placement of cylindrical and tapered dental implants

**DOI:** 10.6026/973206300210884

**Published:** 2025-04-30

**Authors:** Kuldeep Pal, Swati Tiwari, Sumit Patidar3, Rashmi Rai, Anurag Tripathi

**Affiliations:** 1 Department of Oral & Maxillofacial Surgery, Consultant Surgeon, Pal Hospital, Sagar, Madhya Pradesh, India; 2 Department of Oral & Maxillofacial Surgery, Consultant, Dr. Mudgal's Sparsh Hospital and Research, Centre, Gwalior, Madhya Pradesh, India; 3 Department of Oral & Maxillofacial Surgery, Head & Neck Cancer, Reconstructive and Cranio-Maxillofacial Surgeon, Consultant at OraMax Clinic, Indore, Madhya Pradesh, India; 4 Department of Oral & Maxillofacial Surgeon, Consultant Surgical Oncology, Balco Medical Centre, Raipur, Chhattisgarh, India; 5 Department of Oral & Maxillofacial Surgery, Consultant Surgeon, Clinic Oramax Dant Chikitsalaya, Bilaspur, Chhattisgarh, India

**Keywords:** Dental implants, immediate placement, delayed placement, cylindrical implants, tapered implants, implant stability, marginal bone loss

## Abstract

Clinical analysis was performed on both immediate and post-healing implant insertion of cylindrical and tapered dental implants. Hence, 40 patients who required a single implant tooth in the mandibular posterior section were included in this study. Tapered implants yielded the best clinical results, thanks to superior primary stability and minimized marginal bone loss, mainly when used in delayed implantation procedures. The two different implant designs continue to yield successful treatment outcomes when healthcare professionals apply appropriate selection processes and implement proper techniques. The healing response was more favorable in delayed placement procedures compared to immediate placement.

## Background:

Medical science now recognizes dental implants as a dependable tooth replacement option that is widely accepted by people. The clinical achievements of implant therapy result from various contributing elements that combine the timing of placement with bone quality characteristics, surgical procedures and design parameters [1, 2]. Dental professionals used to implement implants after soft and hard tissue regeneration was finalized following tooth extraction [3]. Modern implant placement techniques, combined with advanced implant surface technologies, have made immediate procedures more widely accepted by patients, as they are completed in a shorter timeframe [4, 5]. The dimensions of implant components directly impact primary stability, as they significantly influence the osseointegration process, particularly in situations where bone volume is reduced [6]. Cylindrical implants feature parallel walls to create uniform force distribution; however, they do not effectively grasp the extraction socket edges [7]. The tapered implant design, which replicates root anatomy, provides improved stability by tightly compressing bone tissue and extending more profoundly into the apical bone [8, 9]. The immediate implant procedure benefits significantly from this design since stability achievement remains challenging [10]. Therefore, it is of interest to investigate the clinical performance of cylindrical and tapered implants installed with immediate and delayed protocols, assessing implant stability and examining marginal bone loss and peri-implant soft tissue status.

## Materials and Methods:

The study, which was conducted in a clinical setting, received approval from the Institutional Review Board for its ethical aspects. The research enrolled 40 patients aged 25 to 55 years who required single-tooth implants for their posterior mandibular area. The study selected patients who met several criteria: being systemically healthy with good oral hygiene and having sufficient bone width (at least 8 mm) and height (12 mm or above). Additionally, patients were required to have no active infections in the area surrounding the implant placement site. The study excluded patients who had systemic conditions impacting bone metabolism, together with uncontrolled diabetics, smokers, individuals with para-functional habits and patients with periodontal disease. The study participants were randomly divided into two fundamental groups: the immediate implant placement group, following atraumatic extraction (Group I) and the delayed placement Group II, which received implants after three months. The research groups were divided into two distinct subgroups based on implant type, specifically cylindrical design and tapered design. The study categorizes patients into four subgroups, each comprising ten patients: Group IA (immediate placement with cylindrical implants), Group IB (immediate placement with tapered implants), Group IIA (delayed placement with cylindrical implants) and Group IIB (delayed placement with tapered implants). Standard procedures guided all surgical procedures that used local anesthesia.

The surgeons protected socket integrity throughout the extraction procedure before placing implants directly inside the socket during immediate care intervention. The conventional osteotomy was performed in the healed ridges of patients who received their treatment in the delayed group, following standard drilling procedures. The operators measured implant stability using resonance frequency analysis, with Implant Stability Quotient (ISQ) values recorded at the time of implant placement. Baseline, 3-month and 6-month periapical standardized digital radiographic assessments evaluated marginal bone position. The clinical evaluation of healing soft tissue and postoperative adverse events occurred during patient check-ups. After surgery, the dental provider prescribed antibiotic medication, pain relievers and a chlorhexidine mouth rinse for treatment. The patients received scheduled monitoring sessions to assess tissue healing, implant stability and marginal bone changes after the sutures were removed at week one.

## Results:

The research study involved 40 patients over a six-month period. The integration between implants and bone remained successful throughout the research period, without any implant complications, such as infection or failure. The researchers evaluated the findings using ISQ measurements and assessments of marginal bone loss, as well asoutcomes of soft tissue healing. The primary stability results from tapered implants exceeded those of cylindrical implants when used for both immediate and delayed implant placement cases. The implants in Group IB received tapered placement immediately and measured an average ISQ value of 74.2 ± 2.8. In contrast, Group IA received immediate cylindrical placement and demonstrated an average ISQ value of 71.5 ± 3.1. The delayed tapered group (IIB) recorded the highest ISQ measurement at 76.1 ± 2.5, whereas the delayed cylindrical group (IIA) measured 73.3 ± 2.7. The ISQ measurement showed continuous increment for all treatment groups throughout the research period (Table 1). The follow-up examination revealed Group IIB presented the minimum marginal bone loss (0.9 ± 0.2 mm) opposite to Group IA, which displayed the highest (1.3 ± 0.3 mm). The tapered implant types IB and IIB outperformed the cylindrical implant designs IB and IIA in terms of bone preservation, as shown in Table 2. All participant groups achieved satisfactory healing results without facing significant healing complications. In two patients from the immediate placement group, doctors observed mild inflammation that resolved after standard oral hygiene advice was provided. Soft tissue healing yielded the same results for all four groups, with no statistically significant differences.

## Discussion:

Dental implant success depends on three major components: the timing of implant placement, the design of the implants and the quality of the host bone. Primary stability and marginal bone preservation showed superiority for tapered implants compared to cylindrical implants, regardless of when implant placement occurred. Existing studies support research showing that tapered implants provide superior mechanical support, especially in extraction sites and areas with poorly supported bone [1, 2]. The placement of implants right after tooth extraction remains the standard treatment for reducing treatment duration and maintaining alveolar bone structure and surrounding soft tissues [3]. Primary stability remains challenging to achieve in immediate implant placements, particularly when working with cylindrical implants, as our study results demonstrated poor ISQ values in the immediate cylindrical group. Tapered implants demonstrate superior bone stability and torque during fresh extraction placement because they match the root form and compress cancellous bone near the implant base [4, 5]. Patients who place their implants after the extraction site heals fully experience better implant stability at surgery [6]. The measurements showed that delayed placement of tapered implants resulted in the highest values of the implant stability quotient in our tests. Preliminary studies performed by Esposito *et al.* demonstrated that delayed implant procedures offer better healing circumstances together with decreased procedural complications [7]. The success of dental implants is determined significantly by marginal bone loss values. The researchers found that tapered implants had reduced bone loss compared to cylindrical implants because better stress distribution and smaller bone interface micro-movement occurred [8, 9]. Tapered designs reduce stress concentration levels at the crestal bone while preserving bone [10]. The healing process for soft tissue remained unproblematic across all experimental groups, as no statistical differences were observed between them. Proper surgical procedures and oral hygiene care enable both implant designs to heal successfully with peri-implant tissues [11]. A limited number of cases involving instant implant placement displayed delayed inflammatory responses because of surgical trauma as well as plaque accumulation, according to research in this field [12]. Resonance frequency analysis (RFA) enabled researchers to measure implant stability reliably and non-invasively throughout the study. Multiple research studies have confirmed that RFA is effective for monitoring osseointegration progress over extended periods [13]. The collected ISQ data shows that this method effectively distinguishes between implants with different levels of stability, which depend on their design and placement schedule.

This research study presents essential findings, yet it faces certain limitations. The research included few participants because the follow-up period lasted half a year. Longer-term chronic investigations using larger populations should undertake additional research to validate these findings, as well as assess implant survival and effectiveness rates during extended periods [14, 15]. The research would benefit from a comprehensive implant analysis using three-dimensional imaging combined with biomechanical examination to gain a deeper understanding of implant performance. The research findings receive additional support from studies demonstrating how implant macro-structural elements influence the allocation of force throughout bone and tissue remodelling. The specific design of tapered implants achieves an even distribution of occlusal stress across the entire implant length, thus minimizing local crestal bone damage [16]. The particular design benefit proves most helpful when treating softer bone tissues located in the posterior mandibular regions, which struggle to achieve adequate primary stabilization [17]. The bone compression capabilities of cylindrical implants often fail to meet the same standards as those of these implants, especially during immediate implant procedures. The surface properties of implants should be considered, as their effects have not been directly investigated, yet they may have impacted the trial outcomes. Modern dental implant surfaces that undergo sandblasting combined with acid etching exhibit improved bone-to-implant contact during early osseointegration, as they offer increased roughness, which facilitates better osteoblastic cell activity [18]. Surface modifications found on tapered implants seem to enhance their healing performance during the early healing phase, according to research [19]. Future comparative trials can be improved by investigating different surface treatments in conjunction with implant design features, as the implant surfaces in this research trial employed identical procedures. Patient-related factors such as bone density, oral hygiene compliance and systemic health also play critical roles in the success of implant therapy. Tapered implants demonstrate better predictability in cases of compromised bone density because they offer superior primary stability, according to research [20]. Health providers need to exercise caution about immediate implant placement for patients who carry risks of poor wound healing or peri-implantitis [21]. Treatment plans that utilize digital tools in combination with pre-surgical imaging enhance the accuracy of implant success in these specific cases [22]. Immediate implant placement affects the patient's psychological health together with other treatment factors. Several people opt for immediate surgical interventions because these provide shorter appointment times and eliminate the need for subsequent surgical procedures [23]. The clinician must balance patient optimism with realistic clinical boundaries to determine the suitability of immediate placement, as insufficient bone or infections may render this option inadvisable. The results of long-term implant success hinge on both proper implant selection and a suitable placement approach, alongside effective communication, strong educational efforts and a commitment to maintenance procedures [24, 25].

## Conclusion:

Tapered implants yielded superior results compared to cylindrical implants, particularly in immediate and delayed procedures. The clinical results after delayed placement showed better stability while maintaining more marginal bone than immediate placement. Objective implant success becomes possible through strategic patient selection and proper surgical procedures, regardless of the implant design used.

## Figures and Tables

**Table 1:** Comparison of mean implant stability quotient (ISQ) values across groups

**Group**	**Timing & Implant Design**	**ISQ at Placement**	**ISQ at 3 Months**	**ISQ at 6 Months**
IA	Immediate + Cylindrical	71.5 ± 3.1	73.0 ± 2.9	74.1 ± 2.6
IB	Immediate + Tapered	74.2 ± 2.8	75.8 ± 2.4	76.5 ± 2.2
IIA	Delayed + Cylindrical	73.3 ± 2.7	74.7 ± 2.5	75.2 ± 2.3
IIB	Delayed + Tapered	76.1 ± 2.5	77.4 ± 2.2	78.0 ± 1.9

**Table 2:** Mean marginal bone loss (in mm) at 6-month follow-up

**Group**	**Timing & Implant Design**	**Mean Bone Loss (mm)**
IA	Immediate + Cylindrical	1.3 ± 0.3
IB	Immediate + Tapered	1.0 ± 0.2
IIA	Delayed + Cylindrical	1.1 ± 0.3
IIB	Delayed + Tapered	0.9 ± 0.2
